# Effect of lateral perturbations on psychophysical acceleration detection thresholds

**DOI:** 10.1186/1743-0003-3-2

**Published:** 2006-01-24

**Authors:** Samantha J Richerson, Scott M Morstatt, Kristopher K O'Neal, Gloria Patrick, Charles J Robinson

**Affiliations:** 1 Biomedical Engineering Program, Milwaukee School of Engineering, Milwaukee, WI USA; 2 Research Services, Overton Brooks VA Medical Center, Shreveport, LA, USA; 3 Center for Biomedical Engineering and Rehabilitation Science, Louisiana Tech University, Ruston, LA, USA

## Abstract

**Background:**

In understanding how the human body perceives and responds to small slip-like motions, information on how one senses the slip is essential. The effect of aging and plantar sensory loss on detection of a slip can also be studied. Using psychophysical procedures, acceleration detection thresholds of small lateral whole-body perturbations were measured for healthy young adults (HYA), healthy older adults (HOA) and older adults with diabetic neuropathy (DOA). It was hypothesized that young adults would require smaller accelerations than HOA's and DOA's to detect perturbations at a given displacement.

**Methods:**

Acceleration detection thresholds to whole-body lateral perturbations of 1, 2, 4, 8, and 16 mm were measured for HYAs, HOAs, and DOAs using psychophysical procedures including a two-alternative forced choice protocol. Based on the subject's detection of the previous trial, the acceleration magnitude of the subsequent trial was increased or decreased according to the parameter estimation by sequential testing methodology. This stair-stepping procedure allowed acceleration thresholds to be measured for each displacement.

**Results:**

Results indicate that for lateral displacements of 1 and 2 mm, HOAs and DOAs have significantly higher acceleration detection thresholds than young adults. At displacements of 8 and 16 mm, no differences in threshold were found among groups or between the two perturbation distances. The relationship between the acceleration threshold and perturbation displacement is of particular interest. Peak acceleration thresholds of approximately 10 mm/s^2 ^were found at displacements of 2, 4, 8, and 16 mm for HYAs; at displacements of 4, 8, and 16 mm for HOAs; and at displacements of 8 and 16 mm for DOAs. Thus, 2, 4, and 8 mm appear to be critical breakpoints for HYAs, HOAs, and DOAs respectively, where the psychometric curve deviated from a negative power law relationship. These critical breakpoints likely indicate a change in the physiology of the system as it responds to the stimuli.

**Conclusion:**

As a function of age, the displacement at which the group deviates from a negative power law relationship increases from 2 mm to 4 mm. Additionally, the displacement at which subjects with peripheral sensory deficits deviate from the negative power law relations increases to 8 mm. These increases as a function of age and peripheral sensory loss may help explain the mechanism of falls in the elderly and diabetic populations.

## Introduction

Standing balance is a task that relies on the integration of sensory systems including somatosensory tactile and joint receptors as well as visual and vestibular systems. Deficits in any one of these systems can have an impact on the ability to detect changes in balance, and prevent a slip or fall.

The normal and abnormal functioning of human sensory or control systems can be studied physiologically with large perturbations that are guaranteed to elicit a response; or psychophysically with peri-threshold stimuli that are at the level of sensitivity that barely reach perception. Psychophysical protocols have been very useful in determining perception detection thresholds of many senses including vision, audition, taste, smell, and touch, all of which have led to a better understanding of sensory processing or sensory deficits [1-5]. Similarly, perception thresholds for complex functions that incorporate one or more of these senses can be studied to gain some insight about how these senses are combined or weighted, and decisions are made based upon these inputs.

Generally a subject with "scale" his/her response to a stimulus depending on the total amount of energy in the stimulus [2]. Since both the duration and intensity of a stimulus contribute to the total energy, detection thresholds often exhibit a "trading relationship" between time and intensity. This type of trading relation is linear on a log-log scale over a certain period of time. However, at the point where a relationship deviates from the straight line, a *critical point *is said to have occurred. This critical point usually correlates with a physical inability or change in the physiology of the system [2].

Psychophysical studies of the perception of whole-body motion stimuli are of use when investigating the interaction of the vestibular and tactile sensory systems. Varied accelerations are generally used to measure for motion sensitivity because dynamic motion is primarily sensed by the vestibular apparatus [6]. Previously, linear whole body movement perception has been tested in varied ways.

Benson et al. [7] were one of the first to incorporate psychophysical procedures into testing acceleration thresholds. Using seated subjects, thresholds for acceleration, velocity, and displacement showed subjects were more sensitive to movements in the X (anterior/posterior) and Y(transverse) directions than to the Z (longitudinal) direction. However, thresholds may have been unduly influenced in this study due to the additional proprioceptive input provided to the subject in the seated position. Fitzpatrick and McCloskey [8] used a similar stair-stepping procedure to determine that proprioceptive input from the ankles was the most sensitive measure of motion during low velocity sway (as in quiet standing). Vestibular input was not used unless large disturbances were experienced, leading to the conclusion that normal standing sway was not influenced by the vestibular system.

To compensate for the inherent drawbacks of seated and belt perturbations, current research has moved towards the use of translating platform paradigms. Brown et al. [9] used a hydraulically driven force plate to study postural EMG responses to varying displacements (5 and 15 cm) and velocities (40 and 60 cm/s). In their study, thresholds were not measured, and thus psychophysical procedures were not used. However, the authors did determine that input platform parameters affected the acceleration and deceleration characteristics of the perturbation, and those changes altered the postural response of the subject. Although this platform can be used for these types of larger perturbation studies, the hydraulically driven platform, as well as some other screw-driven platforms, are inadequate for use with psychophysical testing because of additional movement cues provided to the subject as shown by Robinson et al. [10].

Previously, Richerson et al. [11] used the SLIP-FALLS [10] (Sliding Linear Investigative Platform for Assessing Lower Limb Stability) platform, which was specifically built for psychophysical testing, to determine acceleration thresholds for varying anterior and posterior perturbation types. This study determined that acceleration thresholds (and by extension, motion detection) were not significantly different between anterior and posterior translations, or between translations that had a smooth or jerk acceleration profile. However, higher accelerations were needed over shorter perturbations to be detected. Faulkner [12] used the same platform and testing method to measure acceleration thresholds for anterior perturbations of 0.25, 1, 4, and 16 mm in a group of healthy young adults. He found a negative power law trading relationship between acceleration thresholds and movement length, indicating that as movement length increases geometrically, acceleration thresholds decrease geometrically.

Although studies that only look at healthy young adults are useful in determining baseline measurements and in revealing normal postural control strategies, the clinical purpose of balance testing is to predict those that might fall. Maki et al. [13] did some extensive studies of healthy elder adults and concluded that it was lateral stability, and not anterior-posterior stability, that was the best predictor for future risk of falls. By applying direction specific perturbations in both the anterior-posterior and medio-lateral directions, Allum et al. [14] found slowed and reduced EMG responses to lateral motions in healthy elders.

Healthy elderly individuals are not the only group that is at an increased risk for falling. Due to secondary peripheral neuropathy, individuals with diabetes are at a higher risk of falls because of their increased ranges of sway, velocity of sway, and increased movement of the center of mass [15-17]. The peripheral neuropathy is thought to decrease the afferent information available to the CNS, and thus compromise the control of posture [18].

In light of all this current research, this paper will focus on the determination lateral acceleration detection thresholds (defined as the minimum amount of acceleration of a platform over a set displacement) for displacements of 1, 2, 4, 8, and 16 mm, in healthy young adults, and healthy older adults as well as older adults with diabetic neuropathy. Thresholds to small lateral motions will help explain postural stability and control of balance in a way seldom looked at before, using the three different groups will help explore not only the effect of aging on these acceleration thresholds, but also the effect of the loss of sensory information and its repercussions to balance control. It is believed because of aging and loss of sensory information, the magnitude of acceleration necessary to detect motion will increase in the healthy and diabetic elderly subjects.

## Methods

### Subjects

Subjects included 38 older adults over 50 yrs old. Thirteen had a clinical diagnosis of type II diabetes undertaken by their primary physician (mean age = 58.8 yrs, mean weight = 97.1 kg, mean height = 176.3 cm) and 25 did not (mean age = 59.4 yrs, mean weight = 93.6 kg, mean height = 169.2 cm). The majority of the subjects were recruited from within the Veterans Administration (VA) population at the Overton Brooks VA Medical Center (VAMC). Responses from these groups were compared to a younger adult group (age <25, N = 9, mean = 22.9 yrs, mean weight = 74.6 kg, mean height 168.8 cm) that were recruited through advertising at Louisiana Tech University, and tested at the VA Medical Center. The recruiting, screening, testing and informed consent procedures were reviewed and approved by the local VA Institutional Review Board.

### Screening

Subjects recruited for this study underwent visual, vestibular, auditory, musculoskeletal, and cognitive screenings to ensure that no undiagnosed problem existed that would prevent subjects from completing the study. Those with a current or past history of severe heart, circulation, or breathing problems; chronic lower back pain or spasms; deformities of the spine, bones or joints (including advanced arthritis); cerebral stroke, spinal cord injuries or other damage to the nervous system; non-healing skin ulcers; advanced diabetes; current drug or alcohol dependence; or repeated falls were excluded from the study. Individuals taking any prescription medicine to prevent dizziness were also excluded.

Diabetic individuals targeted for this study were those with very early and mild type II diabetes. The diagnosis of diabetes was done by the subject's primary care physician. Targeted recruits had all been diagnosed within the last 10 years. All subjects with diabetes were using either diet or oral medication to manage blood sugar levels and self-reported stable blood sugar levels at the time of testing.

In addition to this screening, all of the older-aged subjects underwent clinical surface nerve conduction studies of the lower extremities performed at the Neurology Service of the Overton Brooks VAMC by a technician under the supervision of a neurologist. Motor (peroneal and tibial nerve) and sensory (sural nerve) nerves were tested bilaterally to ascertain any abnormalities. According to the standards set fourth by the VA Medical Center, normal motor nerve conduction studies have velocities greater than 44 m/s for peroneal nerve, greater than 41 m/s for tibial nerve, and greater than 34 m/s for the sural nerve. These tests found peripheral neuropathies in all 13 diabetics and none of the remaining older aged subjects, who were thus classified as neurologically intact.

### Psychophysical perturbation testing

To perturb the subject's base of support, a novel horizontal translating platform and data collection system (SLIP-FALLS) was used [10]. The dynamics of the perturbation could be completely specified by the investigator. More importantly, the use of non-contact linear motor and air bearing slides essentially eliminated any vibration, obviating a potential cue for movement. This highly-instrumented platform and its controller enabled precise selection of movement profile, including the platform distance and acceleration. A custom LabVIEW™ (v 7.0) program was used to send serial commands to the controller, and also collected AP and ML Centers-of-Pressure (CoP) from the four load cells supporting the platform.

During all testing subjects stood barefoot and blindfolded on SLIP-FALLS. Using an adaptive 2AFC psychophysical protocol [12], the acceleration thresholds for detecting a medio-lateral horizontal translation of the platform at displacements of 1, 2, 4, 8, and 16 mm were found. A 2AFC protocol was used because instructions in a psychophysical paradigm can influence the subject. This paradigm forced the subject to choose in which interval the movement occurred. Headphones provided masking noise (70 dB SPL), and the commands "*Ready*," "*One*," "*Two*," "*Decide*" with the stimulus presented in interval "One" or "Two". After the word *"Decide," *the subject was required to press a handheld button once or twice to signal in which interval (s)he judged that the stimulus occurred. Displacements were ordered randomly and a rest period of 10 to 20 minutes occurred before another run sequence was done at a different displacement value. Within a displacement, movements were randomly assigned to either interval "One" or "Two" ensuring that an equal number of displacements occurred in each interval. Only one threshold estimate was made per displacement per subject. Acceleration profiles of all movements were chosen to be smooth and sinusoidal. Lateral motions were tested only in the direction of hand dominance (all subjects were right-handed and thus all lateral motions were rightward).

To ensure that the accelerations at a given displacement were iterating towards threshold, the Parameter Estimation by Sequential Testing (PEST) algorithm was used [19]. This algorithm determined the amplitude of the next acceleration stimulus as it was iterated towards threshold. The PEST methodology, and our modifications for limits on the number of stimuli presented [12], ensured that all perturbations were near threshold or at least rapidly converging towards threshold values, within a set of trials limited in number to 30 to prevent fatigue [20]. PEST is one of a class of adaptive psychophysical methods in which the task difficulty is changed dynamically to arrive at a desired level of performance [21]. This technique reduces the number of measurements needed to converge to "threshold." Its importance lies in determining a true threshold, and not a certainty level where all responses are correct [20]. Hence, the PEST target probability is set at a level of change rather than a percentage of "correct" responses. For this work, the target probability was set at 79%, which is larger than the 75% generally used in psychophysical procedures [20].

After a threshold was identified, its validity was checked by a second sequence of fixed stimuli tests called peri-threshold reaction time trials. Five trials at threshold and five trials at 125% of threshold were performed. In these trials, the perturbation occurred at any time after the cue "READY." The subject had to press the doorbell transmitter as soon as they detected the perturbation. To make certain that subjects were not pressing at random, two control trials (no movement of platform) were also provided.

### Statistical methodology

Most human reactions and perception thresholds that are measured using psychophysical methodology follow power law relationships that are linear in the log-log domain [2]. Therefore, all thresholds were transformed into the logarithmic domain before any statistical analysis was done. After transformation, all data was tested for normality to ensure the transformation made the data normally distributed. Repeated Measures Two Way ANOVAs were then used to determine difference in acceleration detection thresholds among groups and displacements. One Way ANOVAs were used to determine if randomized order of displacements had an effect on the acceleration threshold and if gender had an effect on acceleration detection thresholds. A regression analysis was also done to determine the negative power law relation between displacement and acceleration detection threshold. To compute these statistics, SigmaStat (v 3.0) was used and the levels of significance for all tests were 0.05.

## Results

### Empirical relationship between lateral acceleration threshold and perturbation displacement

**Figure 1:**
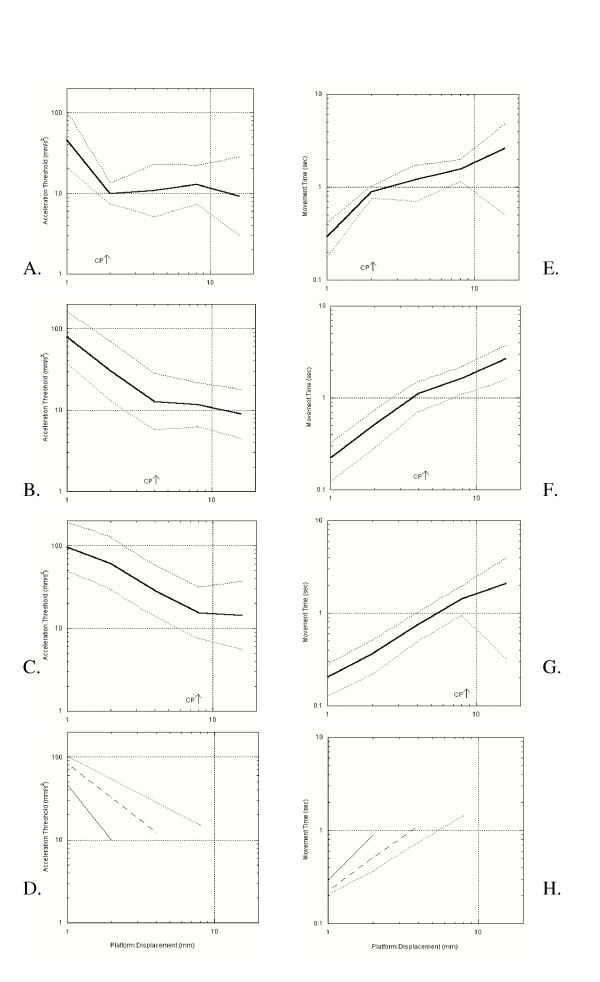
A- D: Geometric Mean and Standard Deviations for lateral acceleration detection thresholds versus Displacement for three groups. Bold lines indicate mean, while thin lines above and below represent the mean +/- 1 geometric standard deviation A: Young adult averages and standard deviations B: Healthy older adults averages and standard deviations C: Diabetic older adults averages and standard deviations. D: Modeled negative power law relationships for healthy young adults (solid line), healthy older adults (long dashed line) and diabetic older adults (short dashed line). Only the linear portion of each curve before the *critical point *was modeled. Thresholds for displacements after the *critical point *were the same in all subjects in all groups (~10 mm/s^2^). E-H: Geometric Mean and Standard Deviation for Movement time versus deisplacement. E: Young adult F: Healthy older adults G: Diabetic older adults. H: Modeled negative power law relationships for healthy young adults (solid line), healthy older adults (long dashed line) and diabetic older adults (short dashed line). Only the linear portion of each curve before the *critical point *was modeled.

The geometric mean and standard deviation of the threshold accelerations for each group were calculated (Table 1). Figure 1a,b,c shows the means (plotted in bold lines) and +/- 1 geometric Standard Deviation (plotted in thin lines), for each of the three groups, young adults (Figure 1a), healthy older adults (Figure 1b), and diabetic older adults (Figure 1c).

**Table 1:** Geometric Means of Lateral Acceleration Detection Threshold with values for average +/- 1 geometric SD in brackets for three groups studied (young adults, healthy older adults, diabetic older adults), at 5 lateral perturbation displacements tested.

Group	N	Mean Age	Threshold at 1 mm (mm/s^2^)	Threshold at 2 mm (mm/s^2^)	Threshold at 4 mm (mm/s^2^)	Threshold at 8 mm (mm/s^2^)	Threshold at 16 mm (mm/s^2^)
Young Adults	11	22.89	46.14^a ^[99.30, 21.30]	9.98^b ^[13.48,7.39]	10.84 [22.94, 5.12]	12.90 [22.36,7.45]	9.28 [28.36,3.03]
Healthy Older Adults	25	59.40	79.37^a ^[166.38,37.86]	30.52^ab ^[70.66,13.18]	12.77 [28.10,6.33]	11.70 [21.60,6.33]	8.92 [17.98,4.43]
Diabetic Older Adults	13	58.85	96.33^ab ^[189.63,48.93]	61.01^ab ^[126.77,29.36]	28.83^ab ^[59.11,14.06]	15.45 [31.83,7.50]	14.44 [37.26,5.60]

a. Indicates significant differences between displacements

b. Indicates significant difference between groups

As can be seen in Table 1 and Figure 1, all groups start with a large acceleration threshold (> 40 mm/s^2^) at small displacements, then at some larger displacement (which is termed the critical point), a minimum in acceleration threshold occurs, followed by a plateau effect. For example, young adults have a high acceleration threshold at 1 mm, a critical point at 2 mm (where threshold is the smallest over all displacements), and after 2 mm (at 4, 8, and 16 mm), all acceleration thresholds are approximately the same (~10 mm/s^2^). The critical point in acceleration threshold occurs at 2 mm for HYA, 4 mm for HOA, and 8 mm for DOA. Plateau acceleration thresholds for each group are approximately the same, again at ~10 mm/s^2^.

A Repeated Measures Two Way ANOVA was used to determine if there were differences in acceleration thresholds across groups or among displacements. Significant differences in acceleration thresholds were seen between groups (dof = 2, F = 9.878, p < 0.001), as well as among displacements (dof = 4, F = 49.221, p < 0.001). The interaction of group and displacement was also significant (dof = 8, F = 2.959, p = 0.004). Pairwise multiple comparison procedures (Tukey's Test) determined that at 1 mm displacements, the acceleration thresholds of HYA are significantly smaller than the acceleration threshold of the DOAs (Diff of means = 0.34, q = 3.2, p = 0.05). However, the acceleration threshold of the HOAs did not differ significantly from the DOAs (Diff of means = 0.079, q = 0.9499, p = 0.78). At 2 mm displacements, DOA had significantly higher acceleration threshold than both HOA's (diff of means = 0.301, q = 3.823, p = 0.019) and HYAs (diff of means = 0.786, q = 7.879, p < 0.001). Additionally, HOAs had a significantly higher acceleration threshold than the HYAs (diff of means = 0.485, q = 5426, p < 0.001). At the 4 mm displacement, DOAs had significantly higher thresholds than both the HOAs (diff of means = 0.354, q = 4.495, p = 0.004) and HYAs (diff of means = 0.425, q = 4.259, p = 0.007), but HYAs and HOAs did not have significantly different thresholds (diff of means = 0.0712, q = 0.796, p = 0.840). At the 8 and 16 mm displacements, no significant differences in acceleration thresholds were seen among groups.

Other experimental factors that may have influenced threshold determination were the order in which the perturbations were presented to the subject (as fatigue is a factor in any balance study), and the gender of the subjects. A One Way ANOVA (dof = 4, F = 0.753, p = 0.568) indicated that the randomized order of displacements did not have an effect on the acceleration detection threshold, which indicates that fatigue was not a factor. Additionally, a One-Way ANOVA showed that there were not any differences in acceleration threshold between gender (dof = 1, F = 1.773, p = 0.184) which indicates that gender did not influence thresholds.

### Negative power law modeling of lateral acceleration threshold verses perturbation displacement

Power law models are commonly used in physiological systems to describe relationships between the intensity of a stimulus and the response of a sensory system [2,3]. In this case, the stimulus was a perturbation of acceleration at a fixed displacement and the response measured was the acceleration detection thresholds. Figure 1a,b and 1c and the results in section IVa show that the three groups tested all performed differently, i.e. 1, 2, 4, 8 mm. A negative power law model was derived for each group over the displacements shown to be significantly different. These models can be seen in Figure 1d.

The solid line in Figure 1d shows the geometric mean of all the HYA subjects. As can be seen from Figure 1a and Table 1, there is a strong negative power law relation for this group over displacements of 1 mm to 2 mm. The steep drop in threshold from 1 mm to 2 mm was significantly different. However, the threshold then levels out at ~10 mm/s^2^, and there is no statistical difference between thresholds at 2, 4, 8, and 16 mm. In the psychophysical realm, this leveling off is called a critical point and indicates a change in the physiology such that the power law relation no longer holds. The possible reasons for this change will be addressed in the discussion. Although it is mathematically unsound to regress with only two points (the R^2 ^value is always 1), the power law relation for young adults can be seen in equation 1 below and will only be used as comparison

Th_a _= 46.136*D^-2.208 ^    (1)

where Th_a _is in mm/s^2 ^and D is in mm.

The long dashed line in Figure 1d shows the geometric mean of all the HOA subjects. The same negative power law trend as the young adults holds, except that in this group, the *critical point *occurs at 4 mm. In the power law region from 1 to 4 mm, the following equation shows the relation of threshold with displacement in the HOA group with an R^2 ^value of 0.9977:

Th_a _= 78.262*D^-1.318 ^    (2)

The short dashed line in Figure 1d shows the geometric mean of all the DOA subjects. In this group, the negative power law relation can be seen over displacements of 1 mm to 8 mm. The *critical point *in diabetic subjects occurs at 8 mm, therefore the power law relation from perturbations from 1 mm to 8 mm yields the following equation with an R^2 ^value of 0.996:

Th_a _= 102.560*D^-0.900 ^    (3)

### Negative power law modeling of lateral acceleration threshold vs perturbation time

The position of the plate and the acceleration of the plate are related by the time of the movement itself using the following equation:



where T is time in seconds, D is displacement in mm, and A is acceleration in mm/s^2^. This equation means that any power law relationship between acceleration and displacement will also result in a power law relationship between acceleration and time. Those relations are shown below and hold over the same displacements as those relations between displacement and acceleration. Power law relations of acceleration threshold with time are also plotted in Figure 1e-g, where the solid line is the mean and the dotted lines are the +/- 1SD line. For young adults, two points were regressed to determine the following power law relation that can be seen in Figure 1e (used only as comparison).

Th_T _= 0.2944*T^1.604 ^    (5)

where Th_T _is in mm/s^2 ^and T is in seconds. For HOA's the power law relation between 1 mm and 4 mm is shown in Figure 2f and in the equation below with an R^2 ^of 0.996:

Th_T _= 0.2261*T^1.159 ^    (6)

For DOA's the power law relation between 1 mm and 8 mm is shown in Figure 1g and in the equation below with an R^2 ^value of 0.998.

Th_T _= 0.1975*T^0.950 ^    (7)

Figure 1h compares the modeled relation between groups. In this plot the HYA's are shown using a solid line, the HOA, a long dashed line, and the DOA's a short dashed line.

## Discussion

### Power law relations between acceleration and displacement

Measurements of acceleration thresholds are a way to determine a subject's sensitivity to motion. It is our contention that the postural control system responds only when exceeding this minimum limit of sensitivity, and that measurement of this lower limit can show insight into how the postural control system comes to attention and initially reacts.

Using similar psychophysical procedures to determine acceleration thresholds of anterior perturbations, Balasubramanian [22] and Faulkner [12] described a negative power law trading relationship between displacement and acceleration for a group of healthy young adults [12], and older adults with and without diabetes [22]. The anterior direction of these perturbations, in conjunction with their small magnitude (0.25 to 16 mm), indicates that an ankle control strategy was predominantly used to react to these perturbations. There was a similar negative power relation between time and anterior acceleration threshold because movement time and displacement were linked. However, it is unknown if the causal variable is time or displacement.

For lateral acceleration thresholds measured here, the inverse relationship between acceleration threshold and displacement is a clear trading relation at displacements less than 8 mm. Additionally, acceleration thresholds yielded group differences that can be used as a balance measure. At small displacements (1 and 2 mm), healthy and diabetic older adults need a higher acceleration to detect motion than young adults, which may be a factor in the higher prevalence of falls seen in these groups.

Clear trading relations were seen in testing, and therefore, power law models were incorporated to further study the relationships. Power law models are commonly used in physiological systems to describe relationships between the intensity of a stimulus and the response of a sensory system. The models developed here show the difference among groups, and indicate that for small perturbations healthy older adults have thresholds that are more than 1.5 times greater than those of young adults. Thresholds of diabetic older adults are 1.3 times greater than healthy older adults, and more than 2 times larger than young adults. It is also apparent from the models that the slopes, or rate of decrease, of acceleration threshold with increased displacement for the different groups are significantly different. Young adults have the largest slope indicating that a small increase in displacement significantly lowers the amount of acceleration necessary for motion detection. The associated decrease in acceleration threshold with an increase in displacement is not as great for either of the other two groups. This may indicate why young adults are better at "catching" themselves after a slip, while healthy and diabetic older adults fall more often.

The critical displacement or breakpoint at which the trading relationship for each group no longer holds is also of interest. Each relationship and critical displacement is dependent upon the group. For young adults, this *critical point *occurs at 2 mm; for healthy adults, 4 mm; and for diabetic older adults, 8 mm. *Critical point *changes occur as a result of a change in physiology of the system [2], and because balance is controlled by restorative torques in the ankles and hips, it is feasible that the *critical point *shows a change in the balance system from an ankle control strategy to a hip control.

In AP perturbations, Winter [23] describes how the CNS stabilizes joints closest to the perturbation first, followed by joints further away, moving up the kinematic chain from ankles, to hips, and finally the spine. This type of response is described as an "ankle strategy". However, in ML directions, Winter describes an alternate strategy, termed a "hip strategy". This strategy claims that ankle muscles are unable to respond because of the positioning of the feet, and instead of the closest joint responding to the perturbation, the hip flexor controls the response to perturbation in lateral directions. This "hip strategy" has been seen in large amplitude (90 mm at a peak acceleration of 1.35 mm/s^2^) studies performed by Henry et al [24,25].

According to Winter, the maximal moment generated about the inverters/everters of the ankles is 10 Nm [23]. Anything over this would cause the foot to roll over; therefore, the hip abductors/adductors generate the needed force to recover from a large moment-generating ML perturbations. However, the reactive force generated by a constant 100 mm/s^2 ^acceleration of a 100 kg person is exactly 10 Nm. The strength of the accelerations presented here indeed fall around this 10 Nm cutoff. Therefore, the restorative force needed to return a person to steady state after a ML perturbation of these small accelerations could be provided entirely via the ankles.

But why should there be differing *critical point*s for different groups? Aging affects balance. Aging has been associated with changes in head and hip sway variability, increases in mean sway in both the AP and ML directions, increases in velocity of sway, and changes in EMG responses of older adults during moderate perturbations [13,26-30]. In addition to changes brought on by normal aging, an aging diabetic subject has even larger sway areas and velocities, and higher thresholds for ankle inversion and eversion [8]. These two increases lead to increased reaction times because the body is forced to rely on the other senses [15,16,31,32]. All of these factors may be part of the reason that the *critical point *occurred at a longer perturbation difference in diabetic subjects than healthy older adults.

### Power law relations between movement time and acceleration threshold

Movement time and displacement are related, therefore, if acceleration thresholds have a power law relation with one of these variables, it must by *de facto *have a power-law relationship with the other. It thus becomes difficult to determine which is the causal partner in the trading relationship with acceleration, even though the experiment was done with the independent variable being displacement.

Many perceptual studies of a variety of sensory systems have shown time to be a trading relationship with psychophysical measures. Block's law is a negative power law trading relationship between the intensity of a visual stimulus and the time that the stimulus is presented [2]. Additionally, Benson et al., fixed the times of linear sigmoidal movements along one of three axes, and found power law trading relationships between peak acceleration and time [7]. In these studies, more intense stimuli required less time to be reliably perceived. This is exactly the case in the experiments reported here. However, looking at the results shown here, it is still unclear if the causal relationship is between time or displacement.

If the acceleration was presented to the subject as an impulse function rather than smoothed as a raised cosine function, then the relationship between time and acceleration would have been linear. If these conditions were to have been met, then the product of acceleration and time would have equaled a fixed velocity, and perception would have simply required that this velocity be exceeded. For this study, impulsive accelerations were purposefully avoided because we felt that it was important to minimize the amount of jerk imposed upon a normal pattern of sway. Further studies are ongoing to look at acceleration and displacement thresholds during constant velocity moves to try and determine the causal element of these relations.

## Conclusion

The acceleration detection thresholds to lateral perturbations measured here are significantly different between young adults, healthy older adults, and diabetic older adults at small (1 and 2 mm) displacements. This shows an affect aging and diabetic neuropathy has on the magnitude of acceleration necessary to perceive a slip of short length. The older individuals needed higher accelerations over short displacements than the young adults to perceive motion. Those individuals with the added deficit of diabetic neuropathy needed even higher accelerations to perceive the same motions. The acceleration detection threshold decreased at even greater displacements which may indicate that a change from ankle strategy to hip stragety in balance control may have occurred. This transition occurred at different displacement lengths for each group and may give some insight to why older adults and adults with diabetic neuropathy have increased risk for slips and falls. Additionally, it has been shown that because there is a power law relation between acceleration threshold and displacement, there is a *de facto *power law relation between acceleration threshold and movement time. Further studies are now underway to determine the causal variable in this relationship.

**Table 2:** Nomenclature

*2AFC*	Two Alternative Forced Choice
*A*	Acceleration
*AP*	Anterior-Posterior
*CNS*	Central Nervous System
*CoP*	Center of Pressure
*D*	Displacement (mm)
*DOA*	Diabetic Older Adult
*HOA*	Healthy Older Adult
*ML*	Medial – Lateral
*PEST*	Parameter Estimation by Sequential Testing
*RL*	Right – Left
*SLIP-FALLS*	Sliding Linear Investigative Platform for Assessing Lower Limb Stability
*T*	Time
*Th*_*a*_	Acceleration Threshold
*HYA*	Young Adult

## Competing interests

The author(s) declare that they have no competing interests.

## Authors' contributions

SR aided study design, data acquisition, as well as completed the data analysis and wrote the manuscript. CJR aided in drafting and revising the manuscript as well as study design.

KO, GP, and SM aided in data acquisition and subject recruitment.
